# Modulation of the Aging Drosophila Brain and Susceptibility to Neurodegeneration

**DOI:** 10.1093/geroni/igaa057.2632

**Published:** 2020-12-16

**Authors:** Nancy Bonini

**Affiliations:** University of Pennsylvania, Philadelphia, Pennsylvania, United States

## Abstract

Human neurodegenerative diseases, like amyotrophic lateral sclerosis, Alzheimer’s disease and frontotemporal dementia, are late-onset progressive neurodegenerative disorders for which few cures or treatments are available. To develop new insight, we have been using the model organism Drosophila. We can re-create disease-associated protein toxicity in the fly brain, and then take advantage of the powerful molecular genetic approaches to define pathways and mechanisms. The fly is allowing us to reveal mechanistic understanding that we can then extend to the human condition. These studies have highlighted multiple novel pathways involved in longterm integrity of the brain. Among these processes, we have defined epigenetic pathways critical for longterm brain health. These pathways typically converge on stress pathway players, highlighting that boosting the ability of the nervous system to combat stress may help promote healthful brain aging and resistance to degenerative disease pathways.

